# When does hepatitis B virus meet long-stranded noncoding RNAs?

**DOI:** 10.3389/fmicb.2022.962186

**Published:** 2022-09-02

**Authors:** Bingxin Lei, Hongxiao Song, Fengchao Xu, Qi Wei, Fei Wang, Guangyun Tan, Haichun Ma

**Affiliations:** ^1^Key Laboratory of Organ Regeneration and Transplantation of the Ministry of Education, Department of Immunology, Center for Pathogen Biology and Infectious Diseases, The First Hospital of Jilin University, Changchun, Jilin, China; ^2^Department of Anesthesiology, The First Hospital of Jilin University, Changchun, Jilin, China

**Keywords:** lncRNA, HBV, interferon, pathogen-associated molecular pattern, IFN-stimulated genes

## Abstract

Hepatitis B virus (HBV) infection in humans and its associated diseases are long-standing problems. HBV can produce a large number of non-self-molecules during its life cycle, which acts as targets for innate immune recognition and initiation. Among these, interferon and its large number of downstream interferon-stimulated gene molecules are important early antiviral factors. However, the development of an effective antiviral immune response is not simple and depends not only on the delicate regulation of the immune response but also on the various mechanisms of virus-related immune escape and immune tolerance. Therefore, despite there being a relatively well-established consensus on the major pathways of the antiviral response and their component molecules, the complete clearance of HBV remains a challenge in both basic and clinical research. Long-noncoding RNAs (lncRNAs) are generally >200 bp in length and perform different functions in the RNA strand encoding the protein. As an important part of the IFN-inducible genes, interferon-stimulated lncRNAs are involved in the regulation of several HBV infection-related pathways. This review traces the basic elements of such pathways and characterizes the various recent targets of lncRNAs, which not only complement the regulatory mechanisms of pathways related to chronic HBV infection, fibrosis, and cancer promotion but also present with new potential therapeutic targets for controlling HBV infection and the malignant transformation of hepatocytes.

## Introduction

### Long-noncoding RNA

In recent years, with increasing understanding of the mechanisms behind gene expression regulation, the role of long-noncoding RNAs (lncRNAs) (please find all of the abbreviations in Supplementary Table S1) in the regulation of signaling pathways has become clear (Sun and Malhotra, 2018; Robinson et al., 2020). Several lncRNAs have been demonstrated to exhibit differential expression in the completed sequencing results of virus-infected cells (Chen et al., 2017; Li et al., 2018a; Gao et al., 2021), signifying their important bidirectional role in viral infection. Initially, lncRNAs were defined as noncoding RNA strands exceeding 200-bp length (Rinn and Chang, 2012). However, some lncRNAs possess the ability to encode small peptides (Ingolia et al., 2011; Guttman et al., 2013); therefore, it is now referred to as long RNA strands that perform a different function than those performed by the encoding proteins (Fitzgerald and Caffrey, 2014). Although this definition distinguishes lncRNAs from mRNAs to the best possible extent, the two types possess several similar properties, most of which are transcribed by the same RNA pol II. Moreover, both share similar processes of post-transcriptional modifications, such as splicing, polyadenylation, and 5′ capsidation (Guttman et al., 2009). Nevertheless, in marked contrast to mRNAs, lncRNAs demonstrate poor sequence conservation and exhibit rapid evolution, although interestingly, their localization in the genome exhibits significant conservation (Ulitsky et al., 2011; Hezroni et al., 2015). This finding suggests that the regulatory role of lncRNA is long-established, yet richly variable, thereby ensuring that the host can cope with diverse pathogenic infections. It is now well established that lncRNAs are involved in several biological functions and disease processes (Ponting et al., 2009; Suarez et al., 2020), but only 4% of all papers on lncRNAs have examined them from an innate immunity perspective (Robinson et al., 2020), making this an area worth exploring.

Because DNA sequences with the protein-coding ability regions have been studied far more deeply and earlier than lncRNAs, the functional mechanisms of several lncRNAs remain unclear. Therefore, several lncRNAs have been named after the nearby protein-encoding genes and are classified according to the positional relationship between them and the nearby related genes, namely sense strand, antisense strand, divergent strand, and intergenic strand. It is therefore convenient to classify known lncRNAs from genetic location, albeit for unknown transcripts, the location of the gene sequence and its coding potential are not precisely linked and still require a multi-perspective experimental approach, such as bioinformatics prediction and sequence co-expression to validate them (Rinn and Chang, 2012). In this process, intergenic lncRNA is clearly the most convenient object to evaluate because various targeting operations on its sequence do not theoretically interfere with the expression of other normal genes in the vicinity. Furthermore, the attribution of the altered biological effects that occur after the targeting operations is more convincing. Considering that location relationships between genes are readily and universally available information when describing transcripts, and the specific biological effects exerted by intergenic strands correspond to the classification of their location; hence, the functional pattern can be predicted to some extent for lncRNAs that have been identified based on localization. The classical lncRNA effects known so far fall into the following 10 main categories: 1. the induction of chromatin remodeling and nucleosome modification; 2. enhancers; 3. mRNA stability; 4. regulation of variable splicing patterns; 5. mRNA editing and capping; 6. generation of endogenous siRNAs; 7. regulation of protein activity; 8. structural or tissue function; 9. alteration of protein localization; and 10. precursors of small RNAs (Wilusz et al., 2009; Hadjicharalambous and Lindsay, 2019).

The expression of lncRNAs is tightly regulated, and they exhibit temporal and spatial specificity of expression (Cabili et al., 2011; Gloss and Dinger, 2016). The expression levels of lncRNAs are extremely variable among different tissues, and, in the same tissues, the abundance is approximately one-tenth of that of mRNA (Pauli et al., 2012). This finding suggests that the regulation of lncRNA expression must be diverse and efficient.

LncRNAs play a bidirectional regulatory role in innate immunity. They can promote the antiviral responses *via* mechanisms, such as the regulation of receptor and signaling pathway key molecular activities (Xie et al., 2018; Xu et al., 2019). These regulatory mechanisms, in turn, inhibit the initiation of immune responses or positive feedback reinforcement (Jiang et al., 2018; Liu et al., 2020). In terms of positional relationship to the regulated gene, both cis- and trans-regulation are present (Engreitz et al., 2016; Yan et al., 2017). Because of the variety and low abundance of lncRNAs, these findings are often based on RNA sequencing. Several sequencing databases are available (Gierahn et al., 2017; Roux et al., 2017), which enrich the sources of lncRNA research.

### Hepatitis B virus life cycle

Hepatitis B virus (HBV)-related diseases are a serious public health concern and pose a huge disease burden. A past study estimated that 2 billion people worldwide were infected by HBV (Schilsky, 2013), of whom 240 million were infected are the hepatitis B surface antigen (HBsAg)-positive chronic carriers (Ott et al., 2012). In most regions, including Asia, the prevalence of HBV infection and associated disease mortality increases significantly with age (Goldstein et al., 2005). In addition, the clinical presentation of HBV infection is varied, and HBeAg-negative patients can be recognized (Zarski et al., 2006). Considering this diversity, in the current context of a progressively aging society, it is imperative to study the relevant properties of HBV in an in-depth manner and conduct drug development research based on it.

At least three types of HBV particles have been reported in the sera of patients with infection: spherical structures of diameter 42 nm, spherical structures of diameter 22 nm, and filamentous structures of variable length and a diameter of 22 nm. Among these, the 42-nm particles, also known as the Dane particles, are infectious viral particles composed of lipid membranes with three HBs antigens, namely large (L-HBs), medium (M-HBs), and small (S-HBs); a nucleocapsid consisting of hepatitis B core protein (HBc); viral polymerase (Pol); and viral genomic DNA surrounded by HBs antigens. The 22-nm particles are abundant in patients’ sera, which include subviral particles (SVP) that lack a nucleocapsid and are, therefore, noninfectious. Other noninfectious particles are currently known to arise from infection, including enveloped particles lacking the viral genome, particles containing viral RNA, and envelope-free particles (bare nucleocapsids; Hu and Liu, 2017; Khuroo and Sofi, 2020). When the infectious viral particles enter the bloodstream, the Supplementary Table S1 prestructural domain of L-HBsAg binds to the sodium taurocholate cotransport polypeptide and induces viral entry into the cells (Yan et al., 2012). Endocytosis is required for entry into the cells, which may be followed by a microtubule-dependent intracellular transport pathway that transports the nucleocapsid to the vicinity of the nucleus (Hayes et al., 2016).

The nucleocapsid is transported to the vicinity of the nuclear pore complex, after which the relaxed circular DNA (rcDNA) is released into the nucleus. Inside the nucleus, rcDNA is converted into covalently closed circular DNA (cccDNA) by a cell-dependent DNA replication mechanism and is subsequently assembled by various protein bindings to form the viral microchromosome (Qi et al., 2016). Multiple factors are involved in the cccDNA formation and maintenance process, albeit the exact mechanisms remain to be elucidated. Immune responses and cytokine stimulation have been reported to be the main factors affecting cccDNA maintenance (Hu et al., 2019). The cccDNA is the main culprit influencing the chronicity of clinical HBV infection and can be stabilized in the nucleus, thereby serving as a template for long-term viral replication. One study estimated the half-life of cccDNA in patients with HBV infection to be >9 months (Boyd et al., 2016). Accurate identification of non-self cccDNA in the nucleus that initiates a strong and persistent response is therefore a crucial host immune response against HBV. The cccDNA is usually assembled with histones and is regulated *via* epigenetic modifications and transcription factors (TFs). There are several research results based on these aspects (Reese et al., 2013; Tropberger et al., 2015); these are important targets for interferons (IFNs) to exert antiviral effects (Liu et al., 2013). Whether lncRNA plays a role in this phenomenon and what molecules and mechanisms are involved in it remain to be investigated in detail.

The cccDNA serves as the template for the viral mRNA library. The virus uses cccDNA to transcribe 3.5, 2.4, 2.1, and 0.7 kb of mRNA. Pregenomic RNA (pgRNA) of 3.5 kb, which can be reverse transcribed from genomic DNA, is used as a template to encode the viral core and polymerase proteins. Pre-c RNA of 3.5-kb size, which encodes HBe, a 2.4-kb RNA, is translated into L-HBs, a 2.1-kb RNA that synthesizes two other surface antigens, M-HBs and S-HBs, and a 0.7-kb RNA that produces HBx (Tsukuda and Watashi, 2020). HBx is a multifunctional protein that promotes viral production in multiple steps, including viral transcription and replication, as well as plays a role in the development of HBV-associated hepatocellular carcinoma (HCC; Murphy et al., 2016). Using cccDNA as a template, viral pgRNA and mRNA encode various viral proteins, including polymerase/reverse transcriptase, nucleoproteins, HBsAg, HBcAg, HBxAg, pgRNA, and Pol, which are wrapped in an icosahedral capsid constituted by HBc to form a nucleocapsid. The RNA within the capsid is reverse transcribed by polymerase/reverse transcriptase using pgRNA as a template to produce rcDNA, which forms the nucleocapsid containing the viral genome. The rcDNA can then be transported back into the nucleus to replenish the cccDNA (recycling) or be secreted outward (efflux; Mohd-Ismail et al., 2019). The exocytosed particles may not contain the full contents of intact HBV particles, which results in the diversity of particles observable in the serum of patients. Dane particles can infect other cells and continue their life cycle (Dane et al., 1970).

**Figure 1:**
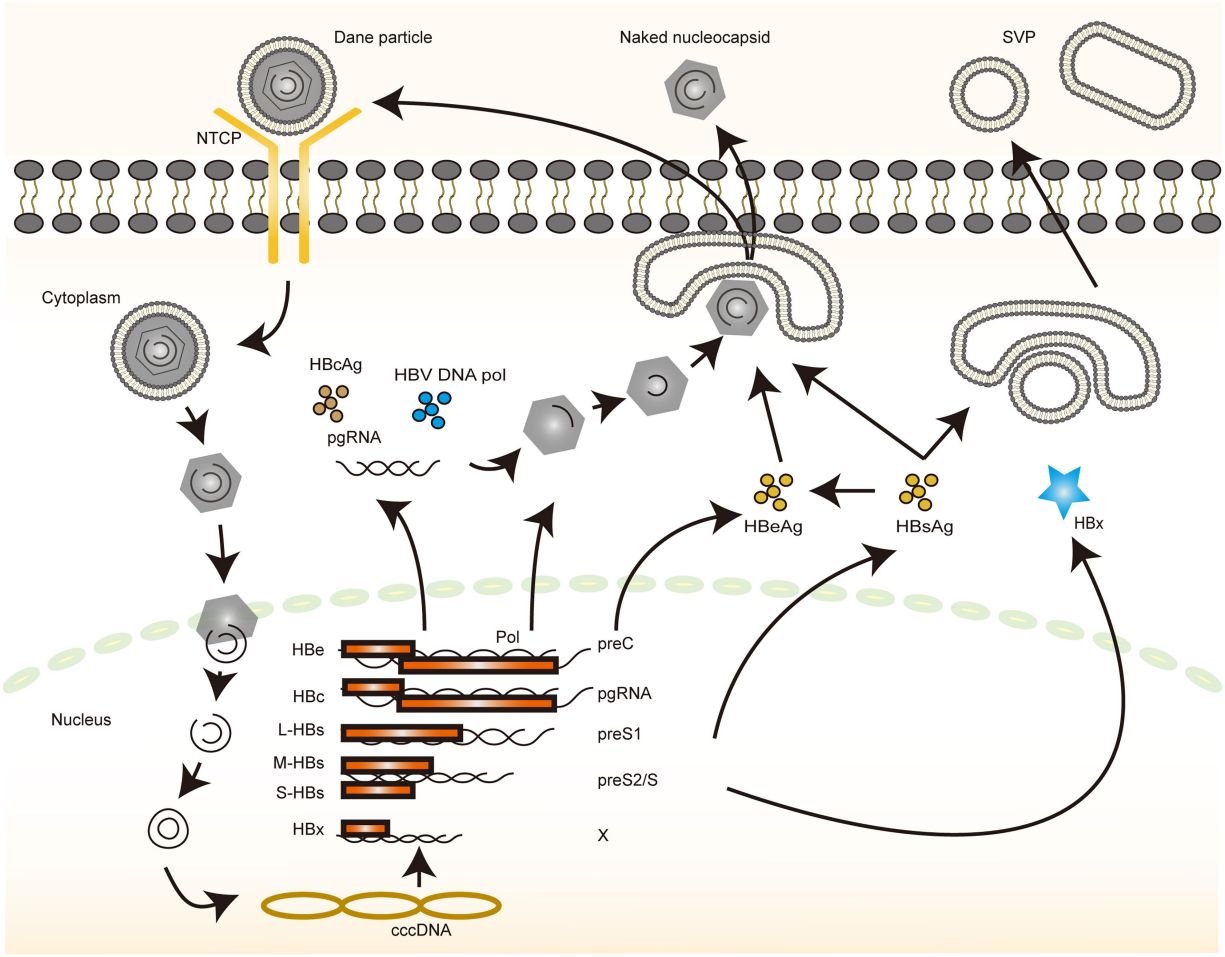
The life cycle of HBV. An illustration depicting the life cycle of HBV. The different color blocks and lines indicate different molecules, and the direction of the arrows indicates the various steps in the process through which HBV completes its replication.

The complex life cycle of HBV involves the conversion of multiple nucleic acid forms and requires several cell-dependent mechanisms as well as specific processes of reverse transcriptional integration, which implies the diversity of interactions with the host (Figure 1). The wealth of regulatory mechanisms enabling the complete clearance of HBV and curing infection remains a clinical challenge. However, with the advent of new technologies, such as novel chimera preparation and high-throughput sequencing, better tools have become available to screen more specific and targeted molecules (Branza-Nichita et al., 2014; Lai et al., 2021). Identifying the different rate-limiting steps in the HBV life cycle and investigating their interaction mechanisms are the key starting points. The early stages of HBV infection contain multiple key steps, and the integration of its genome into the host genome can be completed in a rapid time frame, with the diverse and complex processes providing considerable potential targets for immune system recognition and drug therapy (Herrscher et al., 2020; Pollicino and Caminiti, 2021). However, the immune responses often do not completely clear the virus; hence, characterizing additional molecules and pathways is necessary. N-glycosylation plays multiple roles in the viral life cycle as well as in its interaction with the host; for instance, the three HBV envelope glycoproteins exhibit distinctive N-glycosylation patterns and play important regulatory roles in mediating viral entry into the cells, capsid translocation, and decapsidation (Dobrica et al., 2020).

### Host antiviral response

#### PAMP and PRR

**Figure 2:**
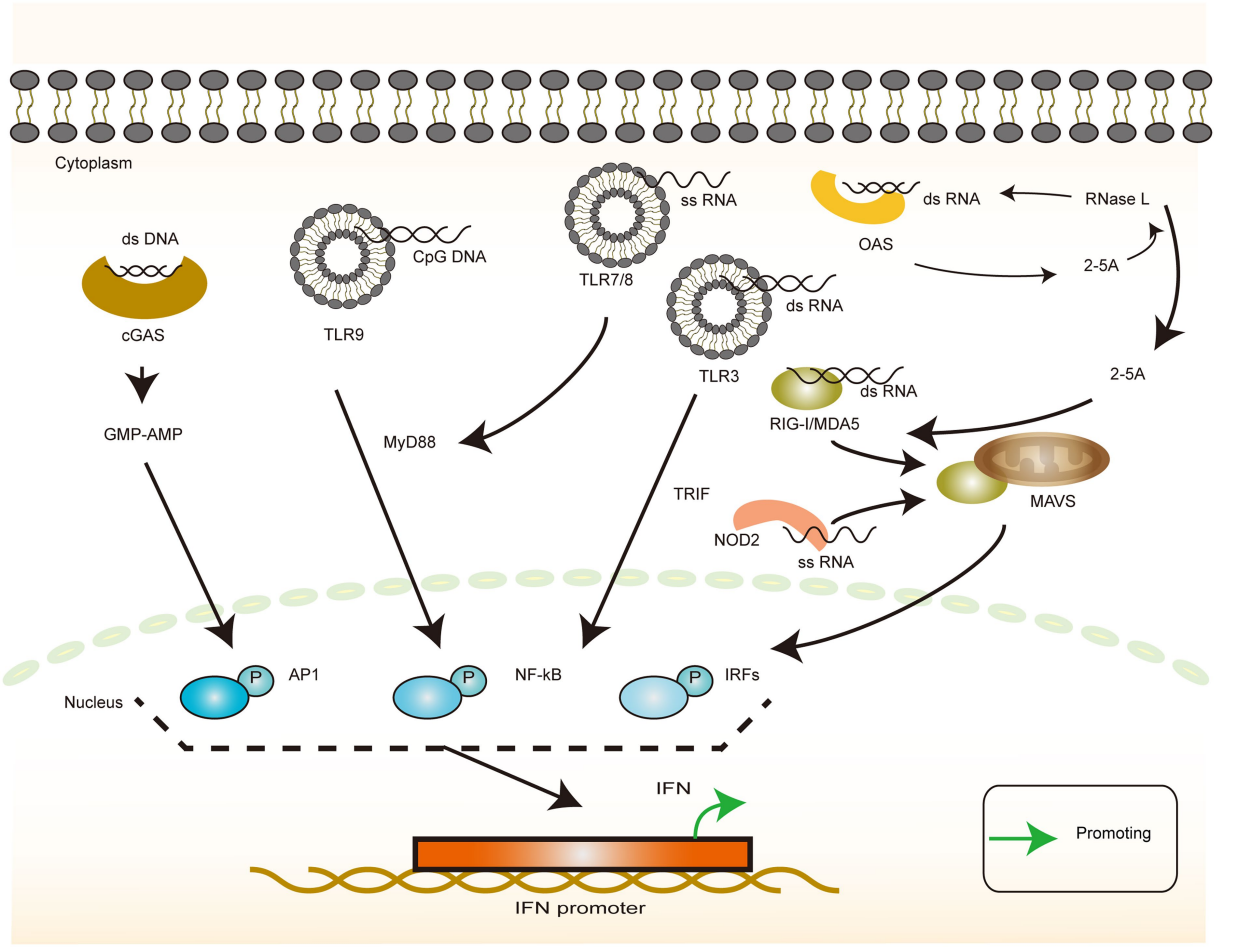
Interferon production in response to heterologous nucleic acid invasion. The illustration of the recognition of different types of viral nucleic acids by host cells and their downstream responses. dsDNA-dependent cGAS induces IFN production via the GMP-AMP pathway. cpGDNA-dependent TLR9 induces IFN production via the MyD88 pathway. dsRNA induces IFN production via the TLR3/TRIF pathway or the RIG-I family/MAVS pathway, or it can be recognized by OAS, producing 2-5A, thereby activating RNA enzymes or activating the intracytoplasmic RNA sensors. ssRNA induces IFN production via the TLR7(TLR8)/MyD88 pathways or NOD2/MAVS pathways.

The immune response against pathogens begins with the nonspecific recognition of the invading pathogen. Pathogen-associated molecular patterns (PAMP) are molecules that are structurally constant and evolutionarily conserved and are not present on the surface of the human host but are shared by several related microorganisms. PAMPs always bind to the pattern recognition receptors (PRRs) and activate a series of intracellular signaling processes, such as the activation of complement, phagocytosis, initiation of cell activation and inflammatory signaling, and induction of apoptosis, which are essential for initiating the immune response. The five common types of PAMP are lipopolysaccharide, lipoprotein, peptidoglycan, lipoteichoic acid, and nucleic acid (Raymond et al., 2017). In the case of HBV, the viral structure does not have such a complex composition of substances, but the basic units of nucleic acid and protein that constitute a virus are necessary for the physiological activity of the host. Accurate and efficient identification, therefore, requires a high degree of precision and flexibility in the regulation of the identification pathway. The nucleic acids available for innate immune recognition can be simply divided into DNA and RNA according to the primary and secondary structure of the recognized nucleic acids, with each member being either single-stranded (ss) or double-stranded (ds) (Chi and Flavell, 2008; Figure 2).

For DNA, toll-like receptor 9 (TLR9) recognizes unmethylated cytosine–guanosine (CpG) sequences in the DNA (Hemmi et al., 2000). This recognition is mainly attributable to the marked differences in the methylation patterns of bacterial DNA and eukaryotic DNA, with the bacterial DNA being methylated mainly on adenine nucleotides and eukaryotic DNA being methylated mainly on cytosine nucleotides. Thus, the CpG fragments are believed to be microbial and can activate TLR9 to, in turn, activate nuclear factor κB (NF-κB) and activator protein 1 *via* the MyD88-dependent pathway in order to regulate the IFN-I expression, particularly the α and β expression (Kawai and Akira, 2007). DNA from viruses is not normally methylated (Kruger et al., 1989). However, after integration into the host genome, methylation of viral DNA resembles that of endogenous eukaryotic DNA (Hoelzer et al., 2008; Bonvicini et al., 2012), which makes identification difficult. Once integrated into the host DNA, the viral DNA remains hidden from the host immune response until the virus gets reactivated. Furthermore, infection of TLR-deficient mice with herpes simplex virus (HSV)-1 controls corneal infection with the virus despite exhibiting an impaired plasmacytoid dendritic cell response (Krug et al., 2004; Mansur et al., 2005). This observation suggests that TLR9 is not the only signaling molecule that mediates the recognition of exogenous DNA. In recent years, cyclic guanosine monophosphate (GMP)-adenosine phosphate (AMP) synthase (cGAS), a novel cytoplasmic dsDNA sensor, has been reported to be a nucleotide transferase that recognizes DNA in a DNA sequence-independent manner. Subsequently, the enzyme converts guanosine triphosphate (GTP) and ATP to the second messenger loop GMP–AMP. This is an endogenous high-affinity ligand for IFN gene stimulators (STING), which recruits and activates TANK-binding kinase 1 (TBK1) and IFN regulatory factor 3 (IRF3) *via* a hyperphosphorylation-dependent mechanism to initiate the transcription of type I IFN and other cytokines (Wu et al., 2013; Shu et al., 2014; Liu et al., 2015a).

For ssRNA, TLR7 and TLR8 are the primary recognition receptors and share a downstream signaling pathway with TLR9. TLR7 and TLR8 exhibit a general specificity for guanosine/uridine-rich ssRNAs and imidazoquinoline-like small synthetic antiviral compounds (Hemmi et al., 2002; Diebold et al., 2004; Heil et al., 2004; Lund et al., 2004). In humans, TLR7 and TLR8 tend to recognize ssRNAs with different sequence characteristics. TLR7 recognizes (in pDC) GU-rich sequences (e.g., UUGU and GUUC) that induce the production of type-I IFN, whereas AU-rich sequences (e.g., AUUU and UAUC) induce the expression of TLR8-mediated TNF responses in monocytes (Forsbach et al., 2008). NOD2 has been reported to be responsible for the recognition of ssRNA and activates IRF3 mainly *via* the MAVS pathway, which, in turn, promotes the IFN expression (Sabbah et al., 2009).

For dsRNA, TLR3 is the primary recognition receptor, and unlike TLR7 and TLR8 that signal *via* MyD88, TLR3 signals *via* TRIF, an adapter containing the TIR domain that induces IFN-β. Binding to ligands recruits TRAF3 and NAP1, which interact with TBK1. Together with IKKε, TBK1 mediates the phosphorylation of IRF3, which is dimerized and translocated to the nucleus (Negishi et al., 2018). TLR3 requires a minimum length of 40 bp for the RNA to be recognized, and the affinity increases with increasing length. However, there is no specific requirement for sequence-specific nucleotide composition (Botos et al., 2009). TLR3 has also been reported to detect viral nucleic acids in infected apoptotic cells after phagocytosis (Schulz et al., 2005). This finding further extends the scope of viral detection. Because dsRNA is a common intermediate in RNA virus replication, TLR3 could act as a more general sensor of viral infection in addition to that of dsRNA viruses (Schulz et al., 2005). MAD5 and RIG-1 can recognize dsRNAs in the cytoplasm of the infected cells. MDA5 preferentially recognizes long dsRNAs >100 bp in length, whereas the RIG-I receptor effectively recognizes sequences as short as 70 bp (Barbalat et al., 2011). The complementary roles of these two recognitions facilitate a mechanism by which the host responds to RNAs of different lengths. In addition, some other types of RNA, such as poly-I:C, 2′,5′-polyadenylate formed *via* RNase L digestion and 5′pppAU-RNA transcribed *via* RNA polymerase III, bind to RIG-I (Hornung et al., 2006; Malathi et al., 2007; Xu et al., 2018). Furthermore, it has been reported that VSV infection induces lncRNA Lsm3b, which acts as a molecular sponge to adsorb and stabilize the RIG-I structure. This stabilization prevents its further activation and the production of IFN (Jiang et al., 2018), thereby inhibiting the positive feedback of IFN production and favoring viral replication.

Moreover, the OAS family of nucleotide transferases, which comprises three homologs encoded by ISG, is activated by virus-produced dsRNA and converts ATP to 2′–5′ oligoadenylate (2–5A). The 2–5A binds to RNase L and triggers its dimerization and activation. RNase L degrades ss viral and cellular RNA, reduces viral replication, and induces apoptosis. RNase L is also a protein that can be induced by the IFN expression, which, in turn, induces the IFN expression *via* MDA5 and induces the expression of IFN, thus creating a positive feedback for the IFN expression. Therefore, the upregulation of the OAS expression enhances the host cell’s antiviral activity (Zhou et al., 1997; Drappier et al., 2014; Drappier and Michiels, 2015). It has been demonstrated that the OASL signaling pathway is distinct in birds and mammals and that the natural switch between OAS/RNase L and OASL/RIG-I signaling is reversible and is mediated by three key D residues in birds and mammals (Rong et al., 2018). This finding explains and unifies the relationship between the two classes of RNA recognition receptors and also reflects the process of host and virus coevolution. As mentioned earlier, there are multiple nucleic acid species and structural transitions in the HBV life cycle, such as the conversion of rcDNA to cccDNA and the reverse transcription of pgRNA, as well as differences in specific mechanisms from normal host nucleic acid metabolism, including DNA damage repair, reverse transcription, and integration. In-depth analysis and mapping of these diversifications and differentiations provide considerable potential targets for immune recognition and drug development (Leong et al., 2015; Tan et al., 2018; Megahed et al., 2020).

#### IFNs pathway

**Figure 3:**
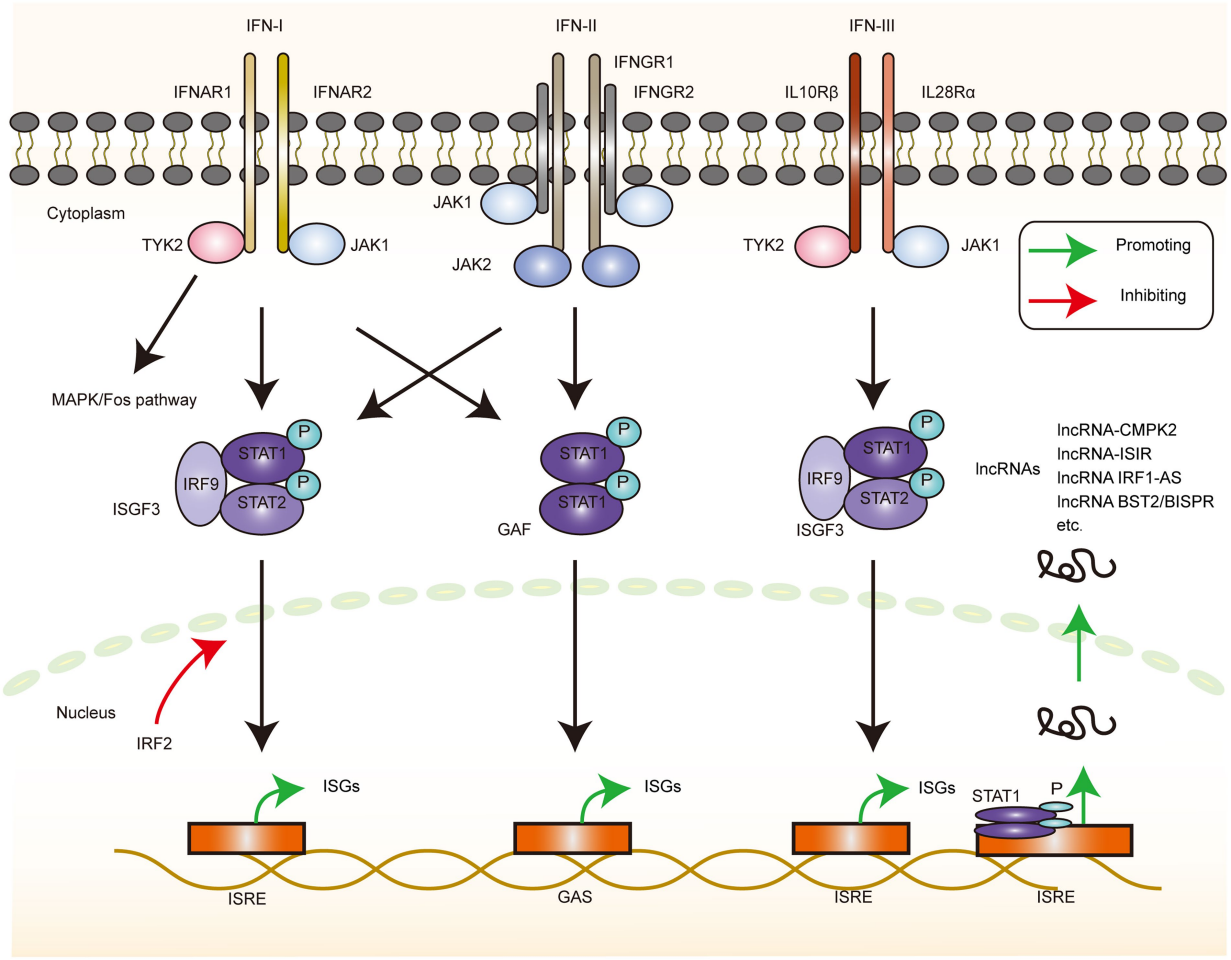
ISGs production via interferon stimulation. The depiction of the downstream signaling pathway changes that occur when the 3 types of interferon receptors bind the corresponding IFN ligands. IFN-I can induce changes in the downstream gene transcription levels via the MAPK/Fos, JAK1-TYK2/ISGF3, and JAK1-TYK2/GAF pathways, with IRF2 serving as a negative regulator of the ISGF3 pathway. IFN-II induces changes in the downstream gene-transcriptional regulation via the JAK1-TYK2/ISGF3 and JAK1-JAK2/GAF pathways. IFN-III produces the downstream gene expression changes via the JAK1-TYK2/ISGF3 pathway. These genes can also be lncRNA, and the current experiments directly or indirectly demonstrated the signaling pathways that upregulate the corresponding lncRNA, mainly through the JAK1/STAT1 pathway, resulting in the production of further biological effects.

After a series of recognition and activation of the signaling pathways, IFNs are produced, which are the early discovered and wide-acting anti-infective factors. IFN derived its name from the ability to interfere with viral replication (Nagano and Kojima, 1958; Weissmann and Weber, 1986; Stark et al., 1998). Type I IFN was discovered first, and approximately two decades later, its gene was successfully cloned and expressed (Maeda et al., 1980; Rubinstein et al., 1981), which contributed immensely to the progress of research in the IFN-related fields. Type II and III IFNs were then discovered subsequently (Wheelock, 1965; Sheppard et al., 2003; Figure 3).

IFN-I is a family of genes, and the human type I IFN family comprises 14 species of IFNα and single species each of IFN-β, IFN-κ, IFN-ω, and IFN-ɛ. Two of these subtypes, α and β, are well studied as they are widely and abundantly expressed. On the contrary, the other subtypes, IFN-ω and IFN-τ, are less well studied because of the limited tissue expression, functional overlap with IFN-α and IFN-β, and interspecies differences (Capobianchi et al., 2015). The downstream signaling of the IFN-I pathway commences with the binding of IFN and the corresponding IFNR, and, after a series of intracellular signaling events, it eventually induces the expression of a series of ISG-based cytokines and related genes to produce the corresponding effects. Specifically, IFN-I induces the formation of the IFNR1–IFNR2 complex, which activates receptor-associated TYK2 and JAK1 kinases. This step is followed by the tyrosine phosphorylation of signal transducer and activator of transcription (STAT)1 and STAT2 as well as the recruitment of IFN regulatory factor 9 (IRF9) to form a heterotrimeric IFN-stimulated gene factor 3 (ISGF3) complex. This complex is then translocated to the nucleus, where it binds to the IFN stimulatory response element (ISRE) in the DNA to initiate gene transcription. This process can be accompanied by the formation of STAT1 homodimers, called IFN-γ activating factors (GAF; Decker et al., 2005). The GAS (IFN-γ activated sequence) elements present in certain ISG promoters and GAF can initiate the transcription of these genes (Platanias, 2005). In addition, IRF2, as a negative regulator of ISGF3-mediated transcriptional activation, controls the intensity of the IFN-I signaling pathway without overreacting (Hida et al., 2000). Type I IFN signaling also activates mitogen-activated protein kinase (MAPK)/p38 and TF c-Fos, independent of the above-mentioned signaling (He et al., 2020). The functioning of this pathway is therefore necessary for the maximum transcriptional activation of IFN-sensitive genes (Platanias, 2003).

IFN-II is an important cytokine secreted by activated NK cells and T cells, but it is structurally unrelated to the other two classes of IFNs (Kasahara et al., 1983; Yu et al., 2006) that bind as homodimers and induce the dimerization of the IFNGR1 subunit and the recruitment of the IFNGR2 subunit. The phosphorylation of the JAK1 and JAK2 kinases leads to STAT1 phosphorylation, and the phosphorylated STAT1 forms the GAF complex. Similarly, IFN-II can also activate ISGF3, although the effect is weak, and the activation of IFN-I signaling can promote effective IFN-II signaling (Takaoka et al., 2000). IFN-II enhances multiple steps in antigen presentation, such as enhancing antigen presentation, promoting helper T cells 1 (TH1) activation to initiate adaptive immunity, clearing infection, and generating memory cells (Schroder et al., 2004). Thus, IFN-II is a key cytokine that links innate and adaptive immunities.

IFN-IIIs (IFN-λs) are structurally related to the IFN-I and IL-10 families (Lazear et al., 2015) and include IFN-λ1 (IL-29), IFN-λ2 (IL-28A), IFN-λ3, and IFN-λ4. Among these, IFN-λ4 was the last to be identified and is a pseudogene in several populations (Prokunina-Olsson et al., 2013). The IFN-III receptor consists of a heterodimer of IL28Rα and IL10Rβ, which then activates the phosphorylation of JAK1-TYK2 kinase, recruits STAT1-STAT2-IRF9 to form ISGF3, and induces the ISG expression, albeit with weak effects (Uze and Monneron, 2007). Despite the receptor differences, the IFN-I and IFN-III downstream signaling pathways and transcriptional responses exhibit a large overlap (Kotenko and Durbin, 2017). However, the biological effects of the two cytokines are not the same. Significant dynamic differences exist in the downstream gene expressions caused by the two cytokines in hepatitis C virus (HCV) infection (Jilg et al., 2014). This difference can be attributed to the fact that HCV evolves multiple inhibitory IFN-I pathways in response to the immune response or to the fact that the long antiviral effect of IFN-III facilitates the complete clearance of the virus and prevents the chronicity of the infection. The activation of IFN-I and IFN-III pathways is also closely related to the distribution of receptors, which may help the body regulate the antiviral responses of cells with different phenotypes (Zhou et al., 2007).

Alterations in the expression of a range of genes can be induced *via* the downstream signaling molecules of IFNs, which are collectively referred to as IFN-inducible genes, representing a collection of IFN-induced molecules with antiviral activity, and their mRNAs, most of which are upregulated following IFN induction. However, some genes are downregulated, and these are often overlooked by researchers. In addition, noncoding RNA (ncRNA) has been identified as an important part of the IFN-inducible genes (Josset et al., 2014; Forster et al., 2015). However, owing to their low abundance and complex and sophisticated regulation of expression, transcriptome analysis (Tong et al., 2016) is a common method for identifying and assessing the potential function and regulation of these ncRNAs. This analysis enables a preliminary classification and exploration of lncRNA through the promoter region accessibility, TF motifs, and histone-modifying activity (Robinson et al., 2020). Transcriptome analysis of IFNα-induced host lncRNAs revealed that IFNα can induce diverse changes in lncRNAs at different time points and that differential lncRNA clusters tend to differ at different time points (Barriocanal et al., 2014; Kambara et al., 2014). This finding suggests that IFN generates a complex network of lncRNA regulation. The proportions of upregulated and downregulated lncRNAs involved in these changes are nearly equal. Most of the lncRNAs are closely related to the neighboring ISGs, which facilitate the predicting of the function of lncRNAs from the perspective of their motif location. Meanwhile, as previous studies have mostly focused on upregulated lncRNAs, these results suggest that downregulated lncRNAs may play an equally important role in the antiviral response. This review aimed to provide a perspective about the lncRNAs’ aspect of the host response to HBV, immune tolerance, and other issues by unraveling and illustrating the mechanisms of lncRNA-related actions linked to the IFN pathway after HBV infection.

## The crosstalk between lncRNA and HBV

### As a TF

**Figure 4:**
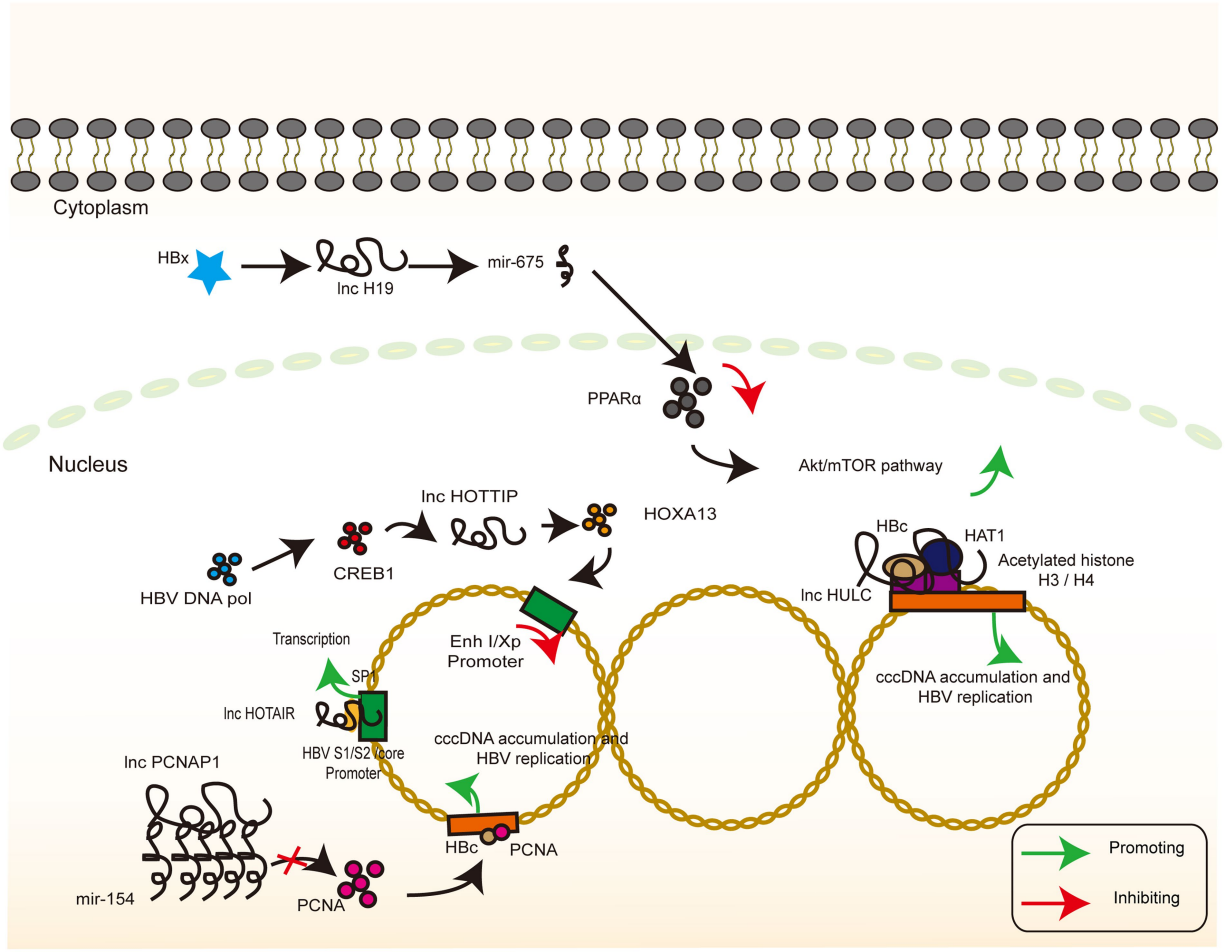
lncRNAs targeting HBV genome. The signaling pathway of IFN-related lncRNA whose target of action is located in the HBV genome. Colour blocks indicate different molecules, irregular lines indicate ncRNA (both lncRNA and miRNA), and large circular lines indicate HBV cccDNA. Green arrows indicate the upregulation, while red arrows indicate the downregulation.

The transcription of cccDNA is a central part of the HBV life cycle. cccDNA transcription products not only direct the synthesis of a series of molecules that eventually assemble into completed HBV viral particles but also integrate with the host genome to maintain chronic infection. Among them, lncRNA HOTAIR, lncRNA HULC, and lncRNA HOTTIP play an important regulatory role in this process. Through the combination of direct effects and indirect regulation of the TF activity, they inhibit the accumulation of cccDNA and HBV replication (Figure 4).

Previous report demonstrated that the HOTAIR overexpression enhanced the luciferase activity of SP1, and lncRNA HOTAIR promotes HBV replication by recruiting SP1 to promote cccDNA transcription (Supplementary Tables S1, S2, and core promoters, not significant for x promoter; Ren et al., 2020). In another study, lncRNA HULC increased the miR-539 promoter activity in the HepG2.2.15 cells. In contrast, silencing of lncRNA HULC decreased the miR-539 promoter activity in the HepAD38 cells, implying that lncRNA HULC can upregulate miR-539 at the transcriptional level. In addition, lncRNA HULC promotes the miR-539 expression by elevating the HBx expression, which further activates STAT3 and binds to the miR-539 promoter. Finally, miR-539 targets the APOBEC3B mRNA 3′UTR and downregulates APOBEC3B, a host factor responsible for the deamination (i.e., destruction) of HBV cccDNA (Zhang et al., 2013; Lucifora et al., 2014), and further leads to an increase in HBV cccDNA as a template for HBV replication (Liu et al., 2019a).

HOXA13 is a molecule downstream of lncRNA HOTTIP that mediates the action of lncRNA HOTTIP on HBV by inhibiting the activity of the HBV promoter EnhI/Xp, as verified by a luciferase reporter assay. Interestingly, HBV DNA pol promotes the expression of CREB1, which, in turn, activates the expression of lncRNA HOTTIP and ultimately inhibits HBV replication (Zhao et al., 2021). Past reports have demonstrated the effects of HBV polymerase in inhibiting the innate immune signaling pathways as well as in mediating immune escape (Yu et al., 2010; Chen et al., 2013; Liu et al., 2014, 2015b). HBV-miRNA-3, also encoded by HBV, targets the ORF of HBV core protein in the 3.5-kb mRNA, which lowers the levels of HBC and pgRNA and attenuates HBV replication (Yang et al., 2017). This negative regulatory pathway in the self-life cycle may facilitate HBV infection, thereby inducing a mild hepatocyte damage and the subsequent establishment and maintenance of chronic infection associated with immune tolerance. However, the exact biological significance of this negative regulatory pathway remains to be elucidated, and neither the relationship with the positive regulatory pathway nor the time point and conditions of activation has been thoroughly investigated.

### Epigenetic modifications

**Figure 5:**
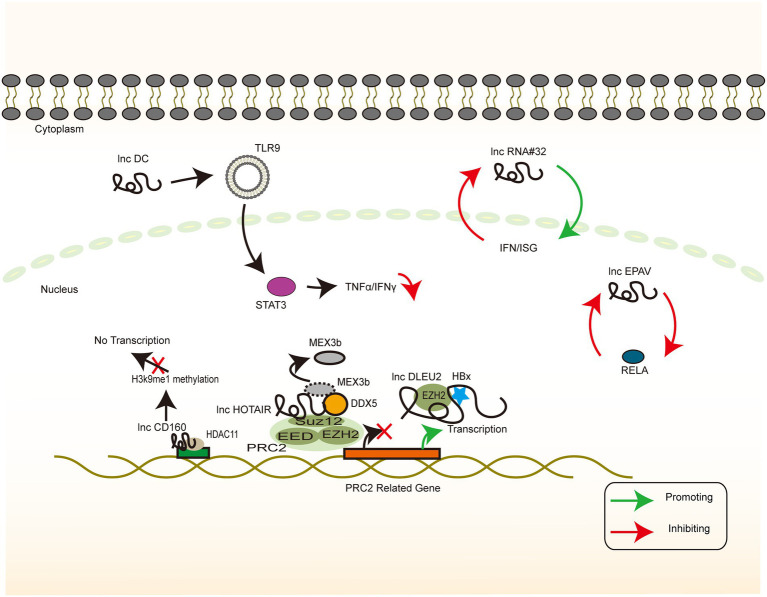
HBV-related lncRNAs targeting the host genome (Part I). The signaling pathway of IFN-associated lncRNA whose target of action is located in the host genome. Colour blocks indicate different molecules, irregular lines indicate lncRNA, and large double-stranded lines indicate host genomic DNA. Green arrows indicate the upregulation, while red arrows indicate the downregulation.

Epigenetic programming is a heritable change in gene regulation that does not involve DNA sequence alteration, and ncRNA-mediated transcriptional and post-transcriptional regulation is an important regulatory mechanism of epigenetic programming (Ma et al., 2014). The partially overlapping reading frame design of HBV allows for a large coding potential of the HBV genome, which is not huge in terms of the base number. To assign the transcriptional process precisely, the precise regulation of the cccDNA microchromosome is warranted, of which histone-mediated transcriptional switches are a common one. lncRNA DLEU2 and lncRNA CD160 both mechanistically regulate gene silencing by altering the methylation modification of the corresponding amino acid residues of histones, with the final effect demonstrating bidirectional antiviral and viral promoting effects. This finding suggests a multifaceted involvement of lncRNA in epigenetic modifications (Bertoletti and Kennedy, 2015; Wu et al., 2020; Figure 5).

It is thus well established that the dysregulation of cellular mechanisms by viral oncoproteins confers a viral growth advantage (Dayaram and Marriott, 2008). HBx has been reported to bind to the lncRNA DLEU2 promoter region, thereby enhancing transcription and inducing its accumulation in the infected hepatocytes. lncRNA DLEU2 binds directly to HBx and zeste homolog 2 (EZH2). Computational models and biochemical evidence suggest that HBx and EZH2 share two preferential binding sites in DLEU2 intron 1. HBx and lncRNA DLEU2 on cccDNA are jointly recruited to displace EZH2 from the viral chromatin in order to promote transcription and viral replication. The association of lncRNA DLEU2-HBx with the target host promoter derepresses EZH2, leading to the transcriptional activation of a subset of EZH2/PRC2 target genes in HBV-infected cells and HBV-associated HCCs (Salerno et al., 2020).

A possible strategy to treat chronic HBV infection is to reverse the immune tolerant phase of patients with CHB infection and to promote the restoration of specific immune responses (Bertoletti and Kennedy, 2015). The staging of HBV infection generally consists of 4 phases: the immune tolerance phase, the immune clearance or active phase, the inactive carrier phase, and the reactivation phase (Trepo et al., 2014). The immune tolerance phase, which is often characterized by normal levels of ALT, low levels of cccDNA, high levels of HBsAg, and low levels of liver inflammation, does not have a consensus definition or management (Terrault et al., 2018). Therefore, an intensive discussion of the mechanisms of its formation and maintenance is essential. From a cellular level perspective, there are significant abnormalities in NK cell function in patients with chronic HBV infection, which may affect the balance of immune factors and T cell activation, and they are thus involved in the establishment and maintenance of immune tolerance (Li et al., 2018b). CD160 binds to MHC class II to mediate the depletion of antiviral CD8+ T cell function, thereby promoting a negative immune regulation (Russell et al., 2017). CD160 negatively regulates HDAC11 and H3K9Me1 in the lncRNA-CD160 motif. lncRNA-CD160 binds to the promoters of IFN-γ and TNF-α *via* HDAC11 recruitment to form a complex that enhances the methylation of H3K9Me1, promotes chromatin heterogeneity, blocks transcription of IFN-γ and TNF-α, and inhibits the transcription of IFN-γ and TNF-α in the CD160-CD8+ T cells. The secretion of IFN-γ and TNF-α further suppresses the CD8 functional + T cells. In animal experiments, serum HBV DNA copy number was found to be significantly lower in LV-lncRNACD160-transfected mice than in LV-control-transfected mice 4–11 days after transplantation. This finding suggests that lncRNA-CD160 plays a key role in the CD8+ T cell immune response to HBV *in vivo* (Wu et al., 2020). The continued exposure of IT-phase T cells to high levels of HBV viral antigens has been reported to be a major cause of T cell depletion and HBV-specific T cells in the hyporeactive state (Fletcher et al., 2013). The elevated levels of CD160 expression have been demonstrated to lead to a high level of HBV replication; this hypothesis explains how the IT phase is maintained and may serve as a therapeutic target worth further exploration.

### As scaffolds or sponges

The regulation of molecular function is not limited to controlling the efficiency of transcription, translation, and degradation but is also common for complex molecules or molecules that change rapidly and over short periods of time. Scaffolds or sponges mechanisms that mask the active sites or direct several molecules to form specific conformations with each other are also a common means of regulation. LncRNAs often act as scaffolds for molecular interactions and are involved in transcriptional regulation (Yang et al., 2013). LncRNA PCNAP1, lncRNA HULC, and lncRNA HOTAIR regulate the activity of target molecules through adsorption and scaffolding, thereby regulating downstream signaling. The exact mechanism of cell-dependent conversion from rcDNA to cccDNA is unclear, but it is generally agreed that the process may be related to DNA damage-repair mechanisms. The involvement of PCNA in the DNA damage-repair mechanism (Moldovan et al., 2007) has been documented. A clear mechanism has been proposed, which was validated through immunoprecipitation (IP), co-IP, and three-dimensional simulated molecular interactions, to exhibit that PCNA is anchored to cccDNA microchromosomes in an HBc-dependent manner, leading to HBV replication and cccDNA accumulation. lncRNA PCNAP1 acts as a molecular sponge that absorbs miR-154 to stimulate the PCNA expression in HCC cells, resulting in cccDNA accumulation and the promotion of HBV transcription and replication (Feng et al., 2019).

Histone modification of viral microchromosomes determines the rate of viral transcription and, in turn, the rate of viral replication (Pollicino et al., 2006). Histone acetyltransferase 1 (HAT1) is responsible for the acetylation of newly synthesized histones and is essential for nucleosome assembly in the host cells. HAT1 is also a key factor in host chromatin assembly (Shahbazian and Grunstein, 2007; Parthun, 2012). HBV can upregulate HAT1 *via* the HBx-controlled TF Sp1. Furthermore, HAT1/CAF-1 signaling, a mechanism of host nucleosome assembly, promotes cccDNA by acetylating histone H4 at the K5 and K12 sites of minichromosome assembly. Moreover, HAT1 is recruited to the cccDNA minichromosome by the lncRNA HULC scaffold HBc, which, in turn, activates HBV transcription and replication (Yang et al., 2019). SUZ12 is the core subunit of PRC2, and its degradation requires the binding of lncRNA HOTAIR, as a ubiquitinated scaffold, to the RNA-binding E3 ligase. DDX5 is an RNA helicase that prevents the degradation of SUZ12 and PRC2 by replacing Mex3b on lncRNA HOTAIR, thereby stabilizing the gene silencing mediated by them (Zhang et al., 2016).

### Immunomodulator

**Figure 6:**
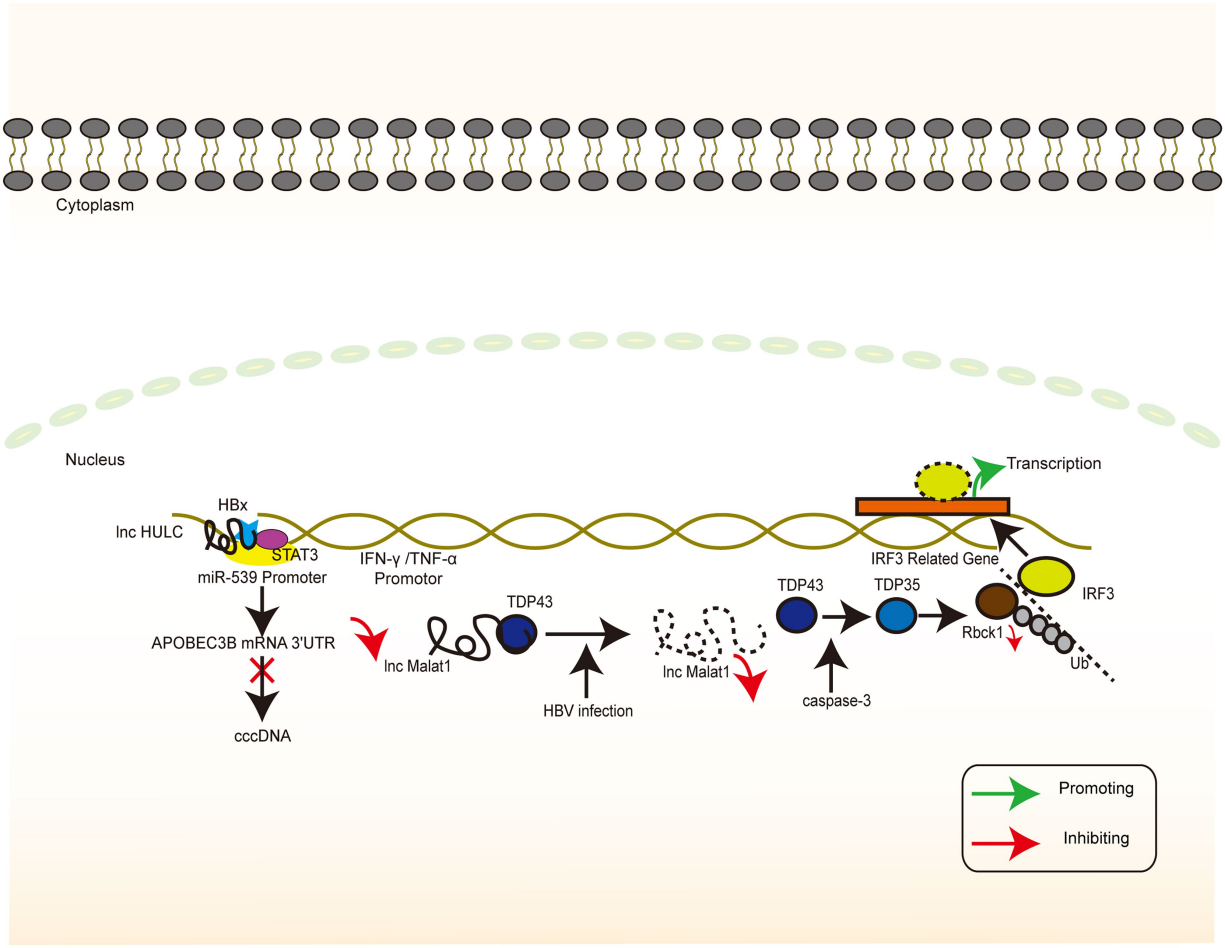
HBV-related lncRNAs targeting the host genome (Part II). The signaling pathway of IFN-associated lncRNA whose target of action is located in the host genome. Colour blocks indicate different molecules, irregular lines indicate lncRNA, and large double-stranded lines indicate host genomic DNA. Green arrows indicate the upregulation, while red arrows indicate the downregulation.

In general, infection-induced IFN responses to HBV tend to be weak and not even induce an innate immune response (Wieland et al., 2004; Suslov et al., 2018), implying that HBV involves a series of mechanisms to evade recognition and suppress the immune response. lncRNA H19, lncRNA DC, lncRNA NEAT1, LncRNA#32, lncRNA Malat1, and lncRNA EPAV regulate the level of innate immune response through interference with the PRR recognition pathway and transcriptional regulation of IFN and ISG. In addition, these mechanisms may form a part of the induction of immune tolerance by HBV (Figure 6). The inhibition of lncRNA H19 or its downstream molecule miR-675 significantly suppresses HBx-induced elevation of a series of immune-related factors represented by IFNs. PPARα and Akt/mTOR signaling are possible downstream molecules and signaling pathways of the above molecules, and their inhibition partially reverses the inhibitory effects of lncRNA H19 and miR-675 (Liu et al., 2019b). The overall effect of lncRNA H19 is favorable for cell injury and the promotion of the immune response to HBV. Although lncRNA H19 is not a molecule encoded by the HBV genome, it can partly explain the intense necrosis of hepatocytes that occurs during severe hepatitis. However, this effect is in contrast to the effect of lncRNA HOTTIP (Zhao et al., 2021), implying that the clinical stage of the HBV infection process represented by acute necrosis and chronic infection involves various complex signaling pathways that mediate different replicative–translational activities, thus presenting with different clinical manifestations. However, the causal relationship between clinical manifestations and signaling pathways warrants further investigation.

HBV activates lncRNA DC in dendritic cells and regulates TLR9/STAT3 signaling. Silencing of lncRNA DC reduces the concentration of TNF-α, IL-6, IL-12, and IFN-γ secreted by dendritic cells and increases the concentration of IL-1β in cells to control the immune response (Zhuang et al., 2018). IFN-γ secreted by dendritic cells plays a key role in Th1 cell activation, which may be related to the difficulty in activating specific T cells in chronic HBV infection. Chronic HBV infection can be caused and maintained by downregulating the functional expression of lncRNA NEAT1, TLR1, TLR6, and RIG-I, which may result in the inability of the cells to produce appropriate signaling molecules to induce the expression of immune factors (Zeng et al., 2019). LncRNA#32 regulates the expression of ISGs (e.g., APOBEC3A and APOBEC3B) and the antiviral effects of type I IFN. This regulation is achieved with the help of heterogeneous nuclear ribonucleoprotein U (hnRNPU) and ATF2, a member of the leucine zipper family of DNA-binding proteins. The suppression of lncRNA#32 by IFN-β treatment may protect the cell from excess inflammation caused by the high expression of ISGs (Nishitsuji et al., 2016). In dormant cells, lncRNA Malat1 binds to TDP43, inhibits IRF3-induced IFN expression, and maintains immune homeostasis. When viral infection occurs, lncRNA Malat1 expression is reduced and, in the absence of lncRNA Malat1 binding, TDP43 is cleaved to TDP35 by activated caspase-3. TDP35 subsequently exerts its antiviral effects by degrading Rbck1 to inhibit its ubiquitination to increase nuclear IRF3 levels and increased IFN production (Liu et al., 2020).

Endogenous retroviruses (ERVs) result from the successful insertion of ancient and modern retroviruses that have become genomically stable and heritable (Johnson, 2015). ERVs exhibit a bidirectional relationship with regard to their action on the host. On the one hand, ERVs may be harmful. On the other hand, ERV sequences or derived molecules may activate the immune system and participate in the regulation of essential immune functions (Chuong et al., 2016). Genome-wide transcriptome analysis using RNA sequencing helped in identifying full-length ERV-derived lncRNA, named lncRNA EPAV (ERV-derived lncRNA positively regulates antiviral responses), as a positive regulator of NF-κB signaling. RELA is an NF-κB subunit that plays a key role in antiviral responses. The lncRNA EPAV coordinates with the transcriptional repressor SFPQ to control the transcription of RELA, while overexpression of RELA can activate lncRNA EPAV transcription, these two form a positive feedback loop (Zhou et al., 2019). The identification of lncRNA EPAV provides evidence for genomic regulation and the strong plasticity of the immune system. Whether this phenomenon is applicable to HBV DNA-derived related ncRNAs remains to be further investigated and provides a possible idea for vaccine development.

### Potential carcinogenic effects

HBV can accelerate HCC *via* multiple mechanisms. To start with, HBV induces an immune response that leads to recurrent liver inflammation, fibrosis, and defects in the immune microenvironment. Furthermore, HBV can modify host genes near the insertion sites *via* DNA integration, which results in host cell genomic instability and the production of oncogenic fusion proteins (Levrero and Zucman-Rossi, 2016). The mouse PVT1 region lies several 100 kilobases (kb) 3′ of the mouse c-myc gene (Huppi et al., 1990). The location of lncRNA PVT1 is thought to be a cancer risk locus shared with the well-known MYC oncogene. Increased lncRNA PVT1 expression is required for MYC protein upregulation in human cancer cells (Tseng et al., 2014). LncRNA PVT1 interferes with EZH2 recruitment to the MYC promoter, which inhibits H3K37me3 modification and promotes hepatitis B c-Myc expression in virus-positive HCC cells. However, whether the upregulation of lncRNA PVT1 is a consequence of HBV infection or tumorigenesis remains elusive (Jiang et al., 2020).

## Concluding remarks

LncRNAs play an important regulatory role in the antiviral response to HBV infection. We summarizes the lncRNAs associated with HBV infection in Supplementary Table S2. With the current progress in gene sequencing and database work, increasing numbers of HBV-associated lncRNAs are being characterized, and further studies on their molecular mechanisms are ongoing. LncRNA regulation of HBV replication and modulating immune response is not limited to interactions with classical factors in the signaling pathways because lncRNAs are also involved in energy metabolism and cellular damage repair (Yu et al., 2018). Owing to the reverse transcriptase profile of HBV, the viral–human gene fusion transcript lncRNA-HBx-LINE1 from genomic integration sites has been found to promote tumor progression (Lau et al., 2014). Furthermore, not all HBV-associated lncRNAs promote HBV replication, which suggests that HBV possesses some mechanisms for replication homeostasis. However, the manner in which this mechanism is regulated warrants further research. The mechanisms of action of lncRNAs are diverse, and because of their noncoding and time–space specificity of expression, they serve as therapeutic targets with a minimal impact on normal cellular metabolism. Recent research on relevant lncRNAs may form the basis for the development of future therapies for viral infections and cancer. The COVID-19 pandemic has accentuated the importance of viral research.

IFN-α has been used to treat HBV, HCV, HIV, HSV, and influenza A virus infections (Enomoto and Nishiguchi, 2015). However, IFNs alone often do not directly inhibit viral replication, and the side effects of IFN therapy can affect several organ systems. Excessive immune response leads to a cytokine storm, which again directly suppresses IFNs and other immune mediators, thereby preventing the healing of the infection. This scenario was encountered in the COVID-19 pandemic. HBV is associated with chronic infection and immune tolerance, wherein the role of CD8+ T cell depletion is largely well understood. However, modulating the activation of this mechanism further and identifying new mechanisms warrant investigations in the future.

During cancer therapy, several lncRNAs act as prooncogenic factors and mediate the maintenance of chronic inflammation and malignant cell appreciation and invasion, thereby promoting tumor progression (Fan et al., 2018; Zuo et al., 2018; Deng et al., 2020; Wang et al., 2021). Moreover, lncRNAs are involved in tumor-associated immune regulation, and the IFN-associated lncRNA INCR1 binds hnRNPH1, thereby preventing it from negatively affecting the expression of neighboring genes PD-L1 and JAK2. This occurrence can lead to increased IFN-γ signaling and affect the CLT-mediated cytotoxic effects against tumor cells (Mineo et al., 2020). The clinical applications of lncRNA-based research are in a stage of rapid development, and there are already examples such as lncRNA HULC and lncRNA MALAT1 (Li et al., 2015; Qiu et al., 2017), which are intensively studied and initially applied as molecular markers. With the continuous expansion of RNA sequencing and databases, lncRNAs are involved in several signaling pathways and play key regulatory molecules. However, there remains a large number of lncRNAs whose roles and mechanisms are unknown and warrant further research. The results from such investigations will provide novel perspectives for an enhanced understanding of the relationship between viral infection and immune response.

## Author contributions

BL drafted the paper. HS, FX, QW, and FW provided suggestions and revised the manuscript. HM and GT revised and wrote the paper. All authors contributed to the article and approved the submitted version.

## Funding

This work was supported by National Natural Science Foundation of China (grant no. 81901592 to HS, 81801565 to FX), the 68th batch of first-class funding from the China Postdoctoral Science Foundation (grant no. 2020M680044 to GT). Jilin University Excellent Young Teacher Training Program to GT.

## Conflict of interest

The authors declare that the research was conducted in the absence of any commercial or financial relationships that could be construed as a potential conflict of interest.

## Publisher’s note

All claims expressed in this article are solely those of the authors and do not necessarily represent those of their affiliated organizations, or those of the publisher, the editors and the reviewers. Any product that may be evaluated in this article, or claim that may be made by its manufacturer, is not guaranteed or endorsed by the publisher.

## Supplementary material

The Supplementary material for this article can be found online at: https://www.frontiersin.org/articles/10.3389/fmicb.2022.962186/full#supplementary-material
