# Supplementary material for: When does hepatitis B virus meet long-stranded noncoding RNAs?
Source: Front Microbiol. 2022 Sep 2;13:962186. doi: 10.3389/fmicb.2022.962186 (PMC9479684; doi:10.3389/fmicb.2022.962186)
Supplement: Supplementary file 1 [file Table_1.DOCX]

**Table 1. List of abbreviations.**

| **Abbreviations** | **Full name** |
| --- | --- |
| HBV | Hepatitis B virus |
| lncRNAs | Long noncoding RNAs |
| IFN | Interferon |
| HBs | Hepatitis B Surface antigen |
| HBc | hepatitis B core protein |
| HBx | hepatitis B X protein |
| Pol | polymerase |
| SVP | subviral particles |
| rcDNA | relaxed circular DNA |
| cccDNA | covalently closed circular DNA |
| pgRNA | Pregenomic RNA |
| PAMP | Pathogen-associated molecular patterns |
| PRRs | pattern recognition receptors |
| ss or ds | single-strandedor or double-stranded |
| TLRx | toll-like receptor x |
| CpG | unmethylated cytosine–guanosine |
| NF-κB | nuclear factor κB |
| MyD88 | Myeloid Differentiation Primary Response Protein 88 |
| HSV | herpes simplex virus |
| GMP | guanosine monophosphate |
| AMP | adenosine phosphate |
| cGAS | cyclic Guanosine monophosphate (GMP)-adenosine phosphate (AMP) synthase |
| GTP | guanosine triphosphate |
| ATP | adenosine triphosphate |
| STING | Stimulator of interferon genes |
| IRF3 | IFN regulatory factor 3 |
| TBK1 | TANK-binding kinase 1 |
| TNF | tumor necrosis factor |
| NOD2 | nucleotide binding oligomerization domain containing 2 |
| MAVS | mitochondrial anti—viral signaling protein |
| TRIF | TIR domain that induces IFN-β |
| TRAF3 | TNF receptor associated factor 3 |
| NAP1 | Nucleosome Assembly Protein 1 |
| IKKε | IkappaB kinase epsilon |
| MAD5 | Melanoma Differentiation-Associated protein 5 |
| RIG-1 | Retinoic acid-inducible gene I |
| Poly I:C | Polysarcoside acid |
| VSV | Vesicular stomatitis virus |
| OAS family | 2 '-5' oligoadenylate synthase family |
| OASL | 2-5-oligoadenylate synthase-like protein |
| ISG | Interferon stimulating gene |
| 2-5A | 2′-5′ oligoadenylate |
| IFNR | Interferon receptor |
| TYK2 | tyrosine kinase 2 |
| JAK1 | Janus kinase-1 |
| STAT1 | signal transducer and activator of transcription 1 |
| ISGF3 | IFN-stimulated gene factor 3 |
| GAF | IFN-γ activated factors |
| GAS | IFN-γ activated sequence |
| IRF9 | IFN regulatory factor 9 |
| ISRE | IFN stimulatory response element |
| MAPK | mitogen-activated protein kinase |
| NK cells | Natural killer cell |
| IFNGR | Interferon gamma receptor |
| IL-10 | Interleukin-10 |
| ncRNA | noncoding RNA |
| TF | transcription factor |
| lncRNA-CMPK2 | Long noncoding RNA ytidine monophosphate kinase 2 |
| lncRNA-ISIR | Long noncoding RNA interferon stimulated IRF3-associating RNA |
| lncRNA IRF1-AS | Long noncoding RNA Interferon Regulatory Factor 1 Antisense RNA |
| lncRNA BST2/BISPR | Long noncoding RNA bone marrow stromal antigen 2/BST2 IFN-stimulated positive regulator |
| SP1 | specificity protein 1 |
| lncRNA HOTAIR | Long noncoding RNA homeobox transcript antisense RNA |
| APOBEC3A/3B | apolipoprotein B mRNA editing catalytic polypeptide-like 3A/3B |
| lncRNA HULC | Long noncoding RNA highly upregulated in liver cancer |
| miR-539 | microRNA-539 |
| 3′UTR | 3' untranslated regions |
| lnc HOTTIP | Long noncoding RNA homeobox transcript at the distal tip |
| HOXA13 | homeobox A13 |
| Enh I/Xp | Enhancer I/ X promotor |
| ORF | Open reading framework |
| CREB1 | cAMP-responsive element-binding protein 1 |
| EZH2 | zeste homolog 2 |
| lncDLEU2 | Long noncoding RNA deleted in lymphocytic leukaemia 2 |
| PRC2 | polycomb repressor complex 2 |
| HCCs | hepatocellular carcinomas |
| CHB | Chronic hepatitis B |
| MHC class II | Major Histocompatibility Complex class II |
| CD160 | Cluster of differentiation |
| HDAC11 | Histone deacetylase 11 |
| H3K9Me1 | Methylation 1 of histone 3 at lysine residue 9 |
| IT-phase | Immune tolerance phase |
| ALT | Alanine aminotransferase |
| LV transfected | Lentivirus transfected |
| PCNA | Proliferating cell nuclear antigen |
| IP | immunoprecipitation |
| lncPCNAP1 | Long noncoding RNA proliferating cell nuclear antigen pseudogenes 1 |
| HAT1 | Histone acetyltransferase 1 |
| CAF1 | chromatin assembly factor 1 |
| SUZ12 | suppressor of zeste 12 homolog |
| Mex3b | M-line-encoding exons 3b |
| PPARα | Peroxisome proliferator-activated receptor-alpha |
| Akt/mTOR signaling | the protein kinase B/mammalian target of rapamycin signaling |
| lnc-DC | Long noncoding RNA dendritic cells |
| lncNEAT1 | Long noncoding RNA nuclear enriched abundant transcript 1 |
| hnRNPU | heterogeneous nuclear ribonucleoprotein U |
| ATF2 | activating transcription factors 2 |
| TDP35/43 | transactive response DNA-binding protein 35/43 |
| lncMalat1 | Long noncoding RNA metastasis-associated lung adenocarcinoma transcript 1 |
| ERVs | Endogenous retroviruses |
| lnc-EPAV | ERV-derived lncRNA |
| SFPQ | splicing factor proline/glutamine rich |
| RELA | reticuloendotheliosis Viral Oncogene Homolog A |
| lncRNA PVT1 | plasmacytoma variant translocation 1 |
| kb | kilobases |
| MYC | myelocytomatosis |
| lnc-HBx-LINE1 | Long noncoding RNA HBV-human chimeric fusion transcript-HBx-long-interspersed nuclear element 1 sequence |
| The COVID-19 | The Corona Virus Disease2019 |
| HBV | Hepatitis B Virus |
| HCV | Hepatitis C Virus |
| HIV | human Immunodeficiency Virus |
| HSV | Herpes Simplex Virus |
| lncRNA INCR1 | Long noncoding RNA IFN-stimulated non-coding RNA 1 |
| hnRNPH1 | heterogeneous nuclear ribonucleoprotein H1 |
| PD-L1 | programmed death ligand1 |
| CLT-mediated cytotoxic | cytotoxic lymphocyte mediated cytotoxic |

This table lists the acronyms covered in the text and their corresponding full names. The acronyms are ordered in the order in which they appear in the text.
