# Supplementary material for: When does hepatitis B virus meet long-stranded noncoding RNAs?
Source: Front Microbiol. 2022 Sep 2;13:962186. doi: 10.3389/fmicb.2022.962186 (PMC9479684; doi:10.3389/fmicb.2022.962186)
Supplement: Supplementary file 2 [file Table_2.docx]

**Table 2. LncRNA associated with HBV infection.**

| **LncRNA** | **Pathway** | **Target** | **Functions** | **Reference** |
| --- | --- | --- | --- | --- |
| lncRNA Lsm3b | nc-Lsm3b competes with viral RNA for binding of RIG-I monomers and blocks downward signaling | RIG-I | Restricting innate immune response | [23] |
| lncRNA HOTAIR | LncRNA HOTAIR recruites SP1 to promote cccDNA transcription | SP1 | Promoting cccDNA transcription | [110] |
| lncRNA HULC | LncRNA HULC promotes miR-539 expression via elevating HBx expression, which further activats STAT3 and binds to the miR-539 promoter. miR-539 targets the APOBEC3B mRNA 3′UTR and downregulates APOBEC3B | HBx/miR-539/ miR-539/ APOBEC3B | Reducing cccDNA degradation | [113] |
| lncRNA HOTTIP | HOXA13, a molecule downstream of lncRNA HOTTIP, binds to and inhibites the activity of the HBV promoter Enh I/Xp. | HOXA13/ Enh I/Xp | Inhibiting HBV replication | [114] |
| lncRNA DLEU2 | HBx and lncRNA DLEU2 on cccDNA are jointly recruited to displace EZH2 from the viral chromatin. | HBx/EZH2 | Promoting viral transcription and viral replication. | [124] |
| lncRNA CD160 | LncRNA CD160 recruites HDAC11 to form a complex that enhances the methylation of H3K9Me1, promoting chromatin heterogeneity, blocking transcription of IFN-γ and TNF-α in CD160-CD8+ T cells. | H3K9Me1/HDAC11/IFN-γ and TNF-α | Conduciving to virus replication. | [121] |
| lncRNA PCNAP1 | LncRNA PCNAP1 absorbs miR-154 to stimulate PCNA expression in HCC cells. | miR-154/PCNA | Promoteing HBV transcription and replication | [132] |
| lncRNA HULC | HAT1 is recruited to the cccDNA minichromosome by the lncRNA HULC scaffold HBc. | HAT1 | Promoteing HBV transcription and replication | [136] |
| lncRNA HOTAIR | DDX5 is an RNA helicase that prevents the degradation of SUZ12 and PRC2 by replacing Mex3b on lncRNA HOTAIR. | DDX5/ Mex3b | Stabilizing the gene silencing mediated by PRC2 | [137] |
| lncRNA H19 | Inhibition of lncRNA H19 significantly suppresses HBx-induced elevation of a series of immune-related factors represented by IFNs. | Maybe signal pathway PPARα and Akt/mTOR | Inhibitsing the expression of interferon and the immune response to HBV | [140] |
| lncRNA DC | LncRNA DC regulates TLR9/STAT3 signaling to control the immune response. | TLR9/STAT3 signaling | May be related to Th1 cell activation | [141] |
| lncRNA NEAT1 | The levels of lncRNA NEAT1 were significantly decreased in CHB patients in the active phase when compared to healthy controls. | Not yet clear | Leading to chronic infection | [142] |
| lncRNA#32 | LncRNA#32 regulates the expression of ISGs and I IFN，with the help of hnRNPU and ATF2 | hnRNPU and ATF2 | Inhibiting the immune response to HBV | [143] |
| lncRNA Malat1 | Malat1 binds to TDP43 to prevent TDP43 activation. Activated TDP43 increases IRF3 protein levels by binding and degrading Rbck1 pre-mRNA. | TDP43/IRF3 | Inhibiting IRF3-induced IFN expression | [22] |
| lncRNA EPAV | LncRNA EPAV is a positive regulator of NF-κB signaling，which coordinates with the transcriptional repressor SFPQ to control RELA transcription | RELA | Promoting the immune response to HBV | [146] |
| lncRNA PVT1 | LncRNA PVT1 interferes with EZH2 recruitment to the MYC promoter, which inhibits H3K37me3 modification and promotes hepatitis B c-Myc expression in virus-positive HCC cells. | EZH2 | Promoting cell malignant transformation | [148] |
| lncRNA INCR1 | LncRNA INCR1 binds hnRNPH1, thereby preventing it from negatively affecting the expression of neighboring genes PD-L1 and JAK2. | hnRNPH1 | Enhanced IFN-γ signaling and affect the CLT-mediated cytotoxic effects against tumor cells | [158] |
| lncRNA HBx-LINE1 | LncRNA HBx-LINE1 promotes activation of Wnt/β-catenin signaling | Wnt/β-catenin signaling | Promoting tumorigenicity | [152] |
| lncRNA MALAT1 and lncRNA HULC | Diverse mechanisms of action | p18/ miR-372/  caspase-3  etc. | As molecular markers in the clinical applications | [160] |

This table summarizes the names of the lncRNAs associated with HBV infection in the article, the main mechanisms and pathways, the main molecules targeted and the final effect on the host cell or HBV.
